# Targeting *Staphylococcus aureus* dominated skin dysbiosis in actinic keratosis to prevent the onset of cutaneous squamous cell carcinoma: Outlook for future therapies?

**DOI:** 10.3389/fonc.2023.1091379

**Published:** 2023-02-02

**Authors:** Jacoba Isobella Bromfield, Philip Hugenholtz, Ian Hector Frazer, Kiarash Khosrotehrani, Janin Chandra

**Affiliations:** ^1^ Frazer Institute, Faculty of Medicine, The University of Queensland, Woolloongabba, QLD, Australia; ^2^ Australian Centre for Ecogenomics, The University of Queensland, St. Lucia, QLD, Australia; ^3^ School of Chemistry and Molecular Biosciences, The University of Queensland, St. Lucia, QLD, Australia; ^4^ Department of Dermatology, Princess Alexandra Hospital, Woolloongabba, QLD, Australia

**Keywords:** *Staphylococcus aureus*, Cutaneous squamous cell carcinoma, Actinic keratosis, *Staphylococcus epidermidis* (*S. epidermidis*), Corynebacterium striatum (*C. striatum*), Cutibacterium acnes (*C. acnes*), UV damage and repair

## Abstract

Cutaneous squamous cell carcinoma (cSCC) and its premalignant precursor, actinic keratosis (AK), present a global health burden that is continuously increasing despite extensive efforts to promote sun safety. Chronic UV exposure is a recognized risk factor for the development of AK and cSCC. However, increasing evidence suggests that AK and cSCC is also associated with skin microbiome dysbiosis and, in particular, an overabundance of the bacterium *Staphylococcus aureus* (*S. aureus).* Studies have shown that *S. aureus*-derived toxins can contribute to DNA damage and lead to chronic upregulation of proinflammatory cytokines that may affect carcinogenesis. Eradication of *S. aureus* from AK lesions and restoration of a healthy microbiome may therefore represent a therapeutic opportunity to alter disease progression. Whilst antibiotics can reduce the *S. aureus* load, antibiotic resistant *S. aureus* pose an increasing global public health threat. The use of specific topically delivered probiotics has been used experimentally in other skin conditions to restore eubiosis, and could therefore also present a non-invasive treatment approach to decrease *S. aureus* colonization and restore a healthy skin microbiome on AK lesions. This article reviews mechanisms by which *S. aureus* may contribute to cutaneous carcinogenesis, and discusses hypotheses and theories that explore the therapeutic potential of specific bacterial species which compete with *S. aureus* in an attempt to restore microbial eubiosis in skin.

## Introduction

Cutaneous squamous cell carcinoma is an invasive keratinocyte cancer arising from the basal layer of the epidermis. A longitudinal analysis of the global burden of cSCC from 1990-2017 revealed a 310% increase in disease incidence, ranking cSCC as the sixth most frequently diagnosed neoplasm worldwide (1). cSCC are thought to result from the malignant transformation of actinic keratosis (AK). AK are characterized as scaly, rough skin lesions, typically less than 1cm in diameter (2). The presence of AK is indicative of cumulative UV-A/B exposure and a lack of regularly implemented sun protection (3, 4). Although the risk of malignant transformation of AK to cSCC is typically 0.0075%/lesion/year, 60% of cSCCs develop from AK lesions (4). AKs that have undergone treatment have a recurrence rate of 20-30% per lesion after 12 months (2, 5). Aside from originating from AKs, an alternative pathway has also been reported where cSCCs arise *de novo* from photo-damaged skin and are not associated with AK (5). A key determinant of progression of AKs or photo-damaged skin to cSCC is immune system competency. Immune suppressed patients, particularly organ transplant recipients, are 200 times more likely to develop cSCC when compared to an immunocompetent age-matched population (6).

cSCC is a multifactorial disease, with the best recognized risk factor being UV exposure, and is endemic amongst Caucasians in tropical and subtropical areas of the globe. UV exposure, particularly UV-B, is associated with epidermal erythema (sunburn), gene mutation, immunosuppression and cancer. The shorter wavelength and higher energy of UV-B enhances damage to DNA and is absorbed in the superficial layers of the skin, mainly the epidermis (7).

Whilst UV exposure contributes to the development of AK and progression of AK to cSCC, it is not currently possible to predict which AK lesions will progress to malignancy. However, 196 genes were found to be differentially expressed between AK and cSCC (8). These genes impact on the mitogen active protein kinase pathways, with overexpression of oncogenes MET, JUN, and PAK2 in cSCC compared to AK, and are associated with loss of differentiation, and gain of malignant properties associated with extracellular matrix remodeling and cell migration (8, 9).

The central dogma of cancer pathogenesis is that all cancers arise as a result of mutated somatic DNA (10). When compared with “normal” skin, skin with solar damage (including AK), skin with selective loss of pigmentation, and aged “normal” skin, each have a substantially higher rate of somatic mutations. These mutations are particularly observed in *TP53*, *CDKN2A*, *KNSTRN*, and *NOTCH1-3* (11–13). NOTCH signaling, for example, is tumor suppressive for squamous cell carcinomas, and loss of this signaling can create a carcinogenic environment that promotes tumor growth (14). When examining the NOTCH genes in particular, Matrincorena et al. found that *NOTCH1* was the most frequently mutated gene in sun exposed skin, with *NOTCH 2* and *NOTCH3* also harboring a significant excess of protein altering mutations (11). Martincorena et al. also identified that normal skin has a high frequency of driver mutations where 20% of cells appearing otherwise normal carried NOTCH mutations, thus indicating that there are other factors that trigger cells to become malignant.

In immune competent patients, the skin has a remarkable ability to combat malignant growth by eliminating abnormal tissue structures (15). Brown et al. demonstrated that mutations in the ß-catenin pathway and Hras^G12V^ in hair follicle stem cells of mouse skin *in vivo* resulted in hair shaft-like outgrowths which extended out through the epidermis and ectopically into the dermis (15). These are known to promote tumorigenic tissue growth in the skin due to an impact on the WNT pathway. However, most of these outgrowths fully regressed within 4 weeks. The study demonstrated that envelopment of the outgrowth by normal cells consistently preceded the expulsion of mutant cells from the tissue, and blocking proliferation of non-mutated cells reduced lesion regression to 2%, compared to the 68% regression in controls. Comparable concepts also applied to mutated stem cells that build the upper epithelium, where large benign tumors regressed over time, and normal tissue architecture and function was re-established. Furthermore, hair follicle ablation was also corrected and restored. It thus appears that the skin is able to correct structural aberrancies caused by gene mutations or physical damage. As the skin has the capacity to eliminate genetically abnormal keratinocytes, there may be other factors besides genetic damage that can promote transformation of AK to cSCC.

### Evidence of the microbiome as a potential driver of skin cancer progression

**Figure 1:**
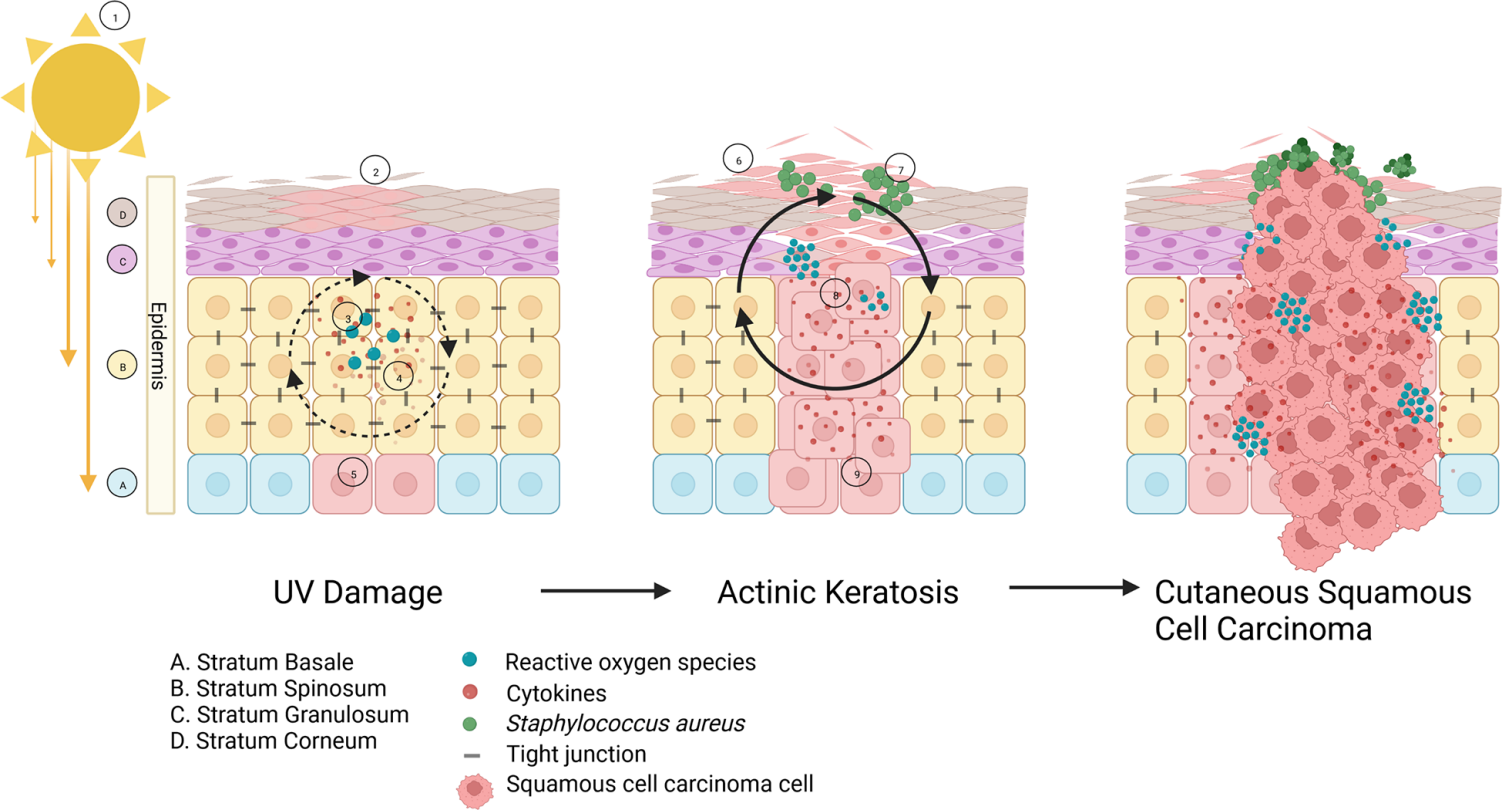
The role of bacteria in AK to cSCC progression: ① Sun overexposure causes UV- A/B radiation to penetrate the epidermis, resulting in ② inflammatory cytokines accumulating in the area and causing erythema. ③ Reactive oxygen species (ROS) are produced and accrue, leading to oxidative stress. ④ The combination of these factors cause ⑤ DNA damage to keratinocytes and ⑥ skin barrier dysfunction including transepidermal water loss and increased pH. As more UV damage to the keratinocytes occurs, the skin barrier function deteriorates and hyperkeratosis can take place to compensate, forming an actinic keratosis (AK) lesion. Meanwhile, a change in skin physiology results in the healthy skin microbiome becoming dysbiotic, and instead enables pathogenic species such as ⑦ *Staphylococcus aureus* to proliferate and release toxins which can induce DNA damage and more ⑧ inflammation and ROS. ⑨ Squamous cell carcinomas begin developing in the basal layer of the epidermis, and penetrate through the stratum corneum creating a cSCC, that can spread into the basal epithelium.

A possible determinant in the progression of AK to cSCC could be changes in the community of microorganisms (bacteria, viruses, and fungi) that reside on the epidermis and in the dermal layers. The skin microbiome assists in maintaining normal skin function by protecting against invading pathogens, interacting with the skin immune system, and processing the breakdown of dead skin cells and other skin products (16, 17). The microbiome of non-lesional photodamaged skin is substantially different from that of AK lesions (18, 19). Sun damage changes the skin in a variety of ways (**Figure 1**). Chronic sun exposure causes inflammation, due to UV-A/B radiation penetrating the skin. This inflammation causes epidermal barrier dysfunction and trans-epidermal water loss, creating the red, dry, and scaly appearance associated with AKs (20). Patients with AK lesions often present with *Staphylococcus* as the dominant taxon on the skin, and a high portion of this taxon are *S. aureus. S. aureus* secrete toxins on the skin which can cause human keratinocytes to overexpress inflammatory factors that may promote skin carcinogenesis (21–24). While no causative relationship between *S. aureus* colonization and AK to cSCC progression has been established to date, this hypothesis presents an interesting interdisciplinary avenue to explore in the fields of oncology and microbiology. Other skin diseases such as atopic dermatitis are also associated with microbial dysbiosis, as the skin’s normal colonizers do not thrive in the altered environment, and pathogens or opportunistic pathogens colonize the affected area. Wood et al. noted that *Malassezia* and *Cutibacterium* (previously known as *Propionibacterium*) are associated with non-lesional photo-damaged skin, whereas *S. aureus* is associated with AK and cSCC lesions (19). A number of studies have suggested a link between the skin microbiota and cSCC progression, whereby *S. aureus* has been found in high relative abundance on AK lesions, but even more so on cSCCs (18, 19, 21–25). The presence of pathogenic bacteria on the skin can cause an inflammatory response, and inflammation is widely recognized as a factor in tumorigenesis, discussed in more detail below (26).

### Relationships between bacterially induced inflammation and tumorigenesis

Skin inflammation can be driven by a variety of factors that ultimately cause leukocytes, phagocytic cells and cytokines to accumulate in skin. Inflammation can induce DNA damage in proliferating cells as it generates reactive oxygen and nitrogen species (ROS and NOS) (27). Typically, in cSCC, UV-A/B exposure acts as an inflammatory stimulus, which causes ROS and NOS to react and form a mutagenic agent, peroxynitrite (27). ROS can cause significant damage to cellular components, including proteins, lipids, and DNA, and lead to apoptotic or necrotic cell death if not neutralized by an antioxidant (28). UV radiation is a potent inducer of ROS, which further accelerates intrinsic and photo-aging of the skin, therefore playing a key role in skin cancer pathogenesis (28). Chronic inflammation has been shown to induce a variety of cancers, including colorectal cancer from inflammatory bowel disease, oesophageal cancer from chronic acid reflux, and bladder cancer from chronic cystitis (29).

Krueger and colleagues investigated the relationship between the secretome of *S. aureus* strains isolated from AK and cSCC lesions, and the induction of pro-inflammatory cytokines produced by keratinocytes (22). Proinflammatory cytokines such as IL-6, IL-8, and TNF-α have previously been shown to promote tumor initiation and progression, and expression of these cytokines is associated with a poor clinical prognosis (30, 31). IL-6 in particular is a key factor for mediating tumor progression. Lederle and colleagues found that IL-6 activates STAT3 and directly stimulates proliferation of benign non-invasive HaCaT-ras A-5 cells *in vitro* (30). IL-6 also leads to overexpression of the collagenase MMP1, thereby enabling migration and invasion. IL-6 further induces a reciprocal cytokine network in tumor tissues, including IL-8, GM-CSF, and VEGF, which supports angiogenesis and leads to the recruitment of immunoregulatory cells that enable tumor progression (30). Krueger and colleagues demonstrated that the cytokines IL-6, IL-8, and TNF-α were overexpressed in HaCaT cells and primary human keratinocytes exposed to filter-sterilized culture supernatants from *S. aureus*, which was consistent with their findings in AK and cSCC biopsies (22). However, clinical isolates of *S. aureus* do not produce a homogenous secretome, and each strain induced a different level of inflammation in keratinocytes. Some *S. aureus* secretomes that were injected into murine skin produced a high level of IL-6 expression intradermally, and resulted in a significant increase in immune cell recruitment, including neutrophils, monocytes, and macrophages, when compared to mice injected with a low-level IL-6 inducing secretome (22). A study conducted by Nakagawa and colleagues investigated the role of the *S. aureus* produced toxin phenol-soluble modulin (PSM)α on inflammation of keratinocytes. They found that this toxin induced the release of various proinflammatory cytokines, including IL-1α and IL-36, and was further associated with the induction of chronically circulating IL-17 (32). IL-17 is a key player in the elimination and protection of cells against bacterial and fungal infection, and its dysregulation is known to be associated with early and late stages of skin cancer. IL-17 signaling also plays a role in wound healing through its association with epidermal growth factor receptor, fibroblast growth factor receptor, and NOTCH1, resulting in keratinocyte proliferation and repair (33, 34). Dysregulation can lead to chronic inflammation and tumorigenesis. These studies demonstrate that *S. aureus* induced inflammation has the potential to contribute to the development of AK lesions and their progression to cSCC.

## Current treatment options for AK and cSCC

Gutzmer and colleagues conducted a comprehensive review on the current treatment options for AK and cSCC, characterizing the AK treatments as ‘lesion-directed’ or ‘field-directed’, and the cSCC treatments as ‘locoregional’ or ‘systemic’ (35). Lesion- directed AK treatments include cryosurgery, ablative and non-ablative laser techniques, and operative techniques (35). While these treatments are effective, some patients cannot undergo surgery due to poor general health, and some surgery causes loss of function, such as reduced eyelid movement, or cosmetic disfigurement to the affected area (36). Patients undergoing these procedures commonly experience pain, bleeding, scarring, erythema, hypopigmentation, and erosions.

The field-directed treatments include topical ointments such as 5-fluorouracil, imiquimod, ingenol mebutate gel, photodynamic therapy, fractional laser resurfacing, and alpha lipoic acid cream (35). The objective of field directed therapies is two-fold. It aims firstly to reduce the number of AKs and to prevent their recurrence. Secondly, it aims to reduce the onset of cSCCs in the future. Among all the immune- modulating and anti-mitotic topical creams only topical fluorouracil has a demonstrated capacity to reduce the risk of cSCC development for 12 months after a 2-4 week course (37). All topical therapies are associated with adverse skin reactions such as erythema, lesion formation, ulceration, and itching (38).

Hence, there is a clinical need for less invasive treatment options for AK that are designed to prevent or reduce the likelihood of progression to cSCC. With increasing recognition of the overall dominant role of the microbiome in health and disease, and a demonstrated microbial dysbiosis on AK and cSCC, it is reasonable to hypothesize that restoration of microbial eubiosis on AK may lead to clinical benefits. While this is an emerging research field, we will here review the current evidence mostly collected from other skin diseases that manipulation of the skin microbiome represents a potential non-invasive method to prevent AK colonization with proinflammatory bacteria that may promote progression of AK to cSCCs.

### Antibiotic therapy

The use of topical antibiotics for skin infections is not a new concept, and common antibiotics such as mupirocin are regularly used for skin infections caused by staphylococci. Skin diseases associated with *S. aureus* colonization including atopic dermatitis (AD) often use this treatment method as colonization with this species results in disease exacerbation and skin barrier dysfunction (39–41). Antibiotics have been shown to decrease the severity of AD, however, there are increasing reports of antimicrobial resistance in *S. aureus* strains isolated from patients with AD (42–44). In particular, methicillin resistant *S. aureus* (MRSA) is becoming increasingly prevalent on children and adults with AD, and skin colonized with MRSA on AD affected sites is associated with a significant decrease in microbial diversity compared to skin colonized with methicillin sensitive *S. aureus* (MSSA) (45). Resistance of *S. aureus* to different antimicrobial agents is typically regional, as different countries have different antibiotic preferences to treat infection (46). Abdulgader and colleagues investigated antibiotic resistance in 212 *S. aureus* isolates obtained from hospital patients over a 6-year period in Cape Town South Africa, and found that 12% were mupirocin resistant, and 44% were MRSA (47). MRSA is a global concern due to the morbidity and mortality rates compared to MSSA, and the increasing prevalence of other antibiotic resistant *S. aureus* should prompt clinicians and researchers to promote and practice good antimicrobial stewardship. Another disadvantage of skin antibiotic treatment is that antibiotics will disturb the entire skin microbiome, while some bacteria are known to play beneficial roles in skin homeostasis. To our knowledge, there are no published studies on the use of antibiotic therapy as part of the management of AK and cSCC. It is likely that such treatments would inhibit *S. aureus* colonization but may also lead to antibiotic resistance and loss of a healthy skin microbiome that may be able to competitively exclude *S. aureus*.

### Bleach baths

The use of sodium hypochlorite (bleach baths) has been a common practice in dermatology to treat AD (48). Similar to AK and cSCC, patients with AD experience an increase in *S. aureus* colonization on the skin associated with increased disease severity and disease exacerbations, and with reduction of commensals such as *Streptococcus, Cutibacterium*, and *Corynebacterium* during flare ups (49). Bleach typically exhibits an effect through non-specific antimicrobial action and is also able to reduce inflammation through inhibition of MAPK and NF-kB signaling (50). The reported effectiveness of bleach baths has been variable, particularly with respect to the reduction of *S. aureus*, likely reflecting variability in the skin microbiome (50–52). Kong et al. found that intermittent bleach treatment significantly reduced *S. aureus* allowing expansion of other bacterial populations and thereby increasing the overall diversity of the skin microbiome (52). Huang et al. found that bleach baths in combination with intranasal mupirocin decreased the *S. aureus* load, and was associated with an improved clinical condition of infection-prone AD patients, as assessed by eczema area and severity index scores (44). However, they also noted that bleach baths did not eradicate *S. aureus.* By contrast, no statistical difference was found between the use of 0.01% bleach baths and water baths with respect to barrier dysfunction, irritation, erythema, transepidermal water loss and pH in both healthy and AD patients (48). Sawada et al. found that bleach baths were only bactericidal at concentrations >0.03%, however this concentration is cytotoxic to human cells and is not clinically recommended (26). Thus, the use of bleach baths at the recommended concentration is safe but clinical outcomes and particularly the effect on *S. aureus* are uncertain.

### Phage therapy

Phage therapy is a century old method used for the treatment of bacterial infection as an alternative to antibiotics (53). Phages are non-living biological entities that are classed as viruses that inject their genetic material into bacterial cells with high host specificity. This can result in the hijacking of the bacterial replication apparatus to produce phage progeny, ultimately resulting in the destruction of the host cell (53). With the rise of antimicrobial resistance, there has been a renewed interest in phage therapy. Phage treatments in experimental mouse infections have demonstrated efficacy and viability for several Gram-negative bacterial infections, including *Acinetobacter baumanii*, *Pseudomonas aeruginosa*, and *Vibrio vulnificus* (54, 55). A study by Capparelli et al. found that the administration of *S. aureus*-targeting phage to mice presented with a lethal dose of *S. aureus* had a 97% rescue rate, and the pathogen was completely eradicated after 4 days of treatment (56). Recently, Shimamori and colleagues demonstrated that a phage isolated from a sewage treatment plant (SaGU1) selectively targeted *S. aureus* and not *S. epidermidis* isolated from the skin of AD patients (57), which is important as the latter is usually considered part of a healthy skin microbiome (see below). However, the authors found that *S. aureus* developed resistance to SaGU1 between 14-24 hours post-inoculation and increasing the concentration of SaGU1 did not decrease the rate of resistance. Interestingly, when combining phage treatment with the secretome of *S. epidermidis*, the *S. aureus* did not regrow, indicating the effectiveness of *S. epidermidis* in combination with phage technology in controlling *S. aureus* colonization (57). However, phage therapy also has several disadvantages, including phages translocating into the blood through the intestinal epithelium when administered orally, which could negatively impact clinical outcome (58). It has also been suggested that phage therapy can induce intestinal barrier dysfunction known as ‘leaky gut syndrome’, which can have serious implications for disorders such as Crohn’s disease, inflammatory bowel disease, and type 1 diabetes (53, 59). This suggests that phage therapy is a potentially viable treatment for *S. aureus* eradication on AK when used in combination with probiotic strains with the additional benefit of not contributing to antimicrobial resistance. An unmet challenge is the provision of standard defined phage preparations, particularly if these are to be used as a regulated therapy.

## Topical probiotics as a potential non-invasive treatment for skin diseases

Alteration of the AK and cSCC microbiome *via* the application of topical probiotics has not previously been attempted. However, there are a number of studies relating to topical probiotics for skin diseases such as AD, acne and eczema that are also characterized by *Staphylococcus* colonization (60). Benefits to local application of probiotics on the skin include competition with harmful skin microbiota, secretion of metabolites, reduction of skin pH, and formation of a barrier or a biofilm to protect the skin from foreign invaders (60). A study conducted by Khmaladze and colleagues applied a topical live probiotic ointment containing *Lactobacillus reuteri* to *ex vivo* skin models and examined the effect of *L. reuteri* on inflammation caused by UV-B radiation (**Table 1**) (61). The authors found that the probiotic application reduced inflammation through reduction of proinflammatory IL-6 and IL-8, whilst also displaying antimicrobial activity against *S. aureus*, among other pathogenic strains. However, *Lactobacillus* strains are sensitive and susceptible to environmental perturbations, particularly heat, and therefore would not have sustainable shelf life. A study conducted by Nakatsuji et al. investigated the variability in coagulase-negative *Staphylococcus* (CoNS) showing antimicrobial activity against *S. aureus* in AD patients and healthy patients, and found that AD patients rarely possess CoNS with antimicrobial activity (62). The authors identified that two CoNS strains, *S. epidermidis* and *S. hominis*, produced strong and selective antimicrobial peptides against *S. aureus* (**Table 1**). The authors isolated three *S. hominis* and two *S. epidermidis* strains from two of five AD subjects, which were formulated into a cream that was applied to AD patients. *S. aureus* abundance on the skin was measured before application, and 24 hours post-application. *S. aureus* was significantly decreased compared to control cream and untreated patients, demonstrating the potential of using topical probiotics to decrease *S. aureus* colonization on the skin. This research indicates that topical probiotics could be advantageous for modulating the AK microbiome, where a particular strain may competitively exclude a harmful pathogen, whilst allowing beneficial and commensal bacteria to recolonize the area.

**Table 1:** Advantages and disadvantages of bacterial species for use as topical probiotics.

	Advantages	Disadvantages
*Staphylococci*-specific antibiotics	Elimination of *S. aureus* on the skin	- Can procure antimicrobial resistance - Allows recolonization of other potential pathogens - Eliminates other *Staphylococcal* species
*Lactobacillus reuteri*	- Decreases inflammation (IL-6 and IL-8) - Antimicrobial activity against *S. aureus*	- Low shelf life and specific storage conditions - Heat sensitive
*Staphylococcus hominis*	- Produces antimicrobial peptides that selectively kill *S. aureus*	- Potential pathogen - Can cause body odor
*Staphylococcus epidermidis*	- Common skin colonizer - Generation of 6-HAP - Generation of Esp that inhibits *S. aureus* biofilm formation	- Opportunistic pathogen in immunocompromised patients - 6-HAP generation is a rare gene not commonly isolated from *S. epidermidis*
*Corynebacterium striatum*	- Decreases agr quorum sensing system - Decreases hemolytic activity - Decreases epithelial cell adhesion	- Does not eliminate *S. aureus*
*Cutibacterium acnes*	- Generation of RoxP resulting in protection of skin against UV-induced free radicals - Common skin colonizer	- Overabundance of *C. acnes* on the face and back can cause acne vulgaris

### Staphylococcus epidermidis

*S. epidermidis* is one of the most common human skin colonizers, and is typically a commensal species in the healthy population that has important functionality for maintaining the skin barrier and integrity (63) (**Table 1**). Nakatsuji et al. found that certain strains of *S. epidermidis* produce a molecule known as 6-HAP, which *in vitro* can selectively inhibit proliferation of tumor lines (64). Additionally, it was found that mice colonized with 6-HAP producing strains had reduced incidence of UV-induced tumors compared to non-6-HAP producing strains. Another potentially important mechanism by which *S. epidermidis* may be able to prevent or reduce *S. aureus* colonization is *via* the protein factor serine protease Esp (65–67). *S. epidermidis* strains containing Esp have been shown to degrade 75 *S. aureus* proteins, 11 of which are needed for biofilm formation and colonization (68). This reduces the viability and pathogenic capability of *S. aureus*, whilst leaving the human host unharmed due to the commensal nature of *S. epidermidis*. However, *S. epidermidis* can be an opportunistic pathogen and cause nosocomial infections from medical devices, particularly in immunocompromised patients (69, 70). Some strains of *S. epidermidis* also possess antimicrobial resistance genes. Therefore it is imperative to evaluate potential strains of *S. epidermidis* to ensure transfer of these resistance genes does not occur (70). Thus, further experimental investigations on utilizing *S. epidermidis* as a topical probiotic for the treatment of AK and cSCC lesions are warranted.

### Corynebacterium striatum

*Corynebacterium striatum* is another commensal bacterium residing on the human skin and on mucosal membranes (**Table 1**). A study published by Ramsey et al. investigated the relationship between *Corynebacterium striatum* and *S. aureus*, and found that *C. striatum* modulated the behavior of *S. aureus* to exhibit commensal rather than pathogenic behavior (71). The analysis revealed that the transcriptome of *S. aureus* changed dramatically, including decreased expression of the accessory gene regulator (agr) quorum sensing system that controls a plethora of virulence factors (71, 72). *S. aureus* also displayed decreased hemolysin activity indicating a reduction in virulence factors, and increased epithelial cell adhesion indicating a commensal state. This could lead to potential treatment options for AKs and cSCCs due to the pathogenic nature of *S. aureus* that has been implicated with the disease. Modulation of the microbial community by means of bacterial behavioral changes rather than bacterial eradication could lead to better patient outcomes cosmetically and functionally due to a sustained healthy skin microbiome physiology.

### Cutibacterium acnes

A commensal species, *Cutibacterium acnes*, is a regular skin colonizer that has demonstrated both beneficial effects and opportunistic pathogenic effects on human skin (73) (**Table 1**). It encompasses 90% of the skin microbiome in predominantly oily areas such as the face and back (74). *C. acnes* has a variety of roles in maintenance of skin homeostasis, including the degradation of long chain fatty acids in sebum to short-chain fatty acids such as propionic acid, which acts as a natural antimicrobial agent on the skin as well as modulating skin pH (74). In terms of the pathogenic effects, *C. acnes* has demonstrated increased abundance on swabs of acne vulgaris and is thought to play a pro-inflammatory role as it is able to form biofilms and change the composition of sebum (74, 75). Whilst not the focus of this review, acne vulgaris is the most frequent diagnosis of skin disease in patients aged 5–44 years, and is an uncommon diagnosis in elderly populations due to the change in the nature of the skin (76). As AK and cSCC are most commonly diagnosed in the elderly population, it is unlikely that a *C. acnes*-based topical probiotic would result in acne vulgaris, however this would need to be investigated further. However, when investigating AD, a lack of *C. acnes*, and its inverse relationship with *S. aureus*, has been identified as a potential contributor to AD pathogenicity, as it has been suggested that patients with AD are deficient in the antimicrobial peptides produced by skin cells (62, 77). *C. acnes* demonstrates a high level of antioxidant activity due to the production of the Radical oxygenase of *Propionibacterium acnes* (RoxP), which appears to be a common property of this species (78). Of particular interest is the relationship between RoxP, UV radiation and cSCC, as RoxP has been shown to protect skin cells against UV radiation by preventing free radical generation (73, 79). A study conducted by Andersson and colleagues studied protein abundance on AKs and found that the concentration of RoxP and *C. acnes* was significantly lower on AK compared to healthy skin (73). The use of bacteria that produce a potent antioxidant would be highly beneficial in AK and early cSCC treatment as it could provide ongoing protection against UV-A/B radiation, as well as neutralizing free radicals and thereby decreasing the overall cellular damage.

## Conclusion and future directions

There is mounting evidence that the progression of UV damaged skin to AK and cSCC is multifactorial. Well recognized factors include UV-A/B radiation and mutational burden associated with age, immune competency, and environmental factors. The role of pathogenic bacteria, specifically *S. aureus*, and microbial dysbiosis in this progression is under active investigation. Although the relationship between *S. aureus* and common skin conditions have been reported in the literature, the effect of *S. aureus* on AK progression to cSCC are not yet fully established. However, recent studies have demonstrated that *S. aureus-*derived toxins can induce tumor-promoting cytokines and reactive oxygen species.

While future studies are needed to determine the magnitude of contribution of microbial dysbiosis to AK and cSCC development, the ability to change the microbial profile by means of restoring a healthy skin microbiome and eliminating pathogenic bacteria, specifically *S. aureus*, has the potential to change the way that premalignancies are treated, and could also lead to new treatment methods for cSCC. While there will be challenges in preparing standardized bacterial products to use as therapy in research studies, the hypothesis that a healthy immune-supportive microbial community stabilized on AKs and cSCCs may result in improved patient outcomes and lower the rate of disease progression is warranted.

## Data availability statement

The original contributions presented in the study are included in the article/supplementary material. Further inquiries can be directed to the corresponding author.

## Author contributions

JIB wrote the first draft of the manuscript. JIB, PH, IF and JC conceptualized the review. JIB, PH, IF, JC and KK revised and edited the manuscript.
